# Anchoring skeletal muscle development and disease: the role of ankyrin repeat domain containing proteins in muscle physiology

**DOI:** 10.3109/10409238.2010.488217

**Published:** 2010-06-01

**Authors:** Jin-Ming Tee, Maikel P. Peppelenbosch

**Affiliations:** 1 Hubrecht Institute for Developmental Biology and Stem Cell Research-University Medical Center Utrecht, Uppsalalaan 8, 3584 CT, Utrecht, The Netherlands; 2 Department of Gastroenterology and Hepatology, Erasmus MC-University Medical Center Rotterdam, 3015 CE, Rotterdam, The Netherlands

**Keywords:** Ankyrin and SOCS-box containing protein, ankyrin repeat, cell differentiation, muscle metabolism, mechanical stress, notch receptor, protein–protein interaction, transcriptional responses

## Abstract

The ankyrin repeat is a protein module with high affinity for other ankyrin repeats based on strong Van der Waals forces. The resulting dimerization is unusually resistant to both mechanical forces and alkanization, making this module exceedingly useful for meeting the extraordinary demands of muscle physiology. Many aspects of muscle function are controlled by the superfamily ankyrin repeat domain containing proteins, including structural fixation of the contractile apparatus to the muscle membrane by ankyrins, the archetypical member of the family. Additionally, other ankyrin repeat domain containing proteins critically control the various differentiation steps during muscle development, with Notch and developmental stage-specific expression of the members of the Ankyrin repeat and SOCS box (ASB) containing family of proteins controlling compartment size and guiding the various steps of muscle specification. Also, adaptive responses in fully formed muscle require ankyrin repeat containing proteins, with Myotrophin/V-1 ankyrin repeat containing proteins controlling the induction of hypertrophic responses following excessive mechanical load, and muscle ankyrin repeat proteins (MARPs) acting as protective mechanisms of last resort following extreme demands on muscle tissue. Knowledge on mechanisms governing the ordered expression of the various members of superfamily of ankyrin repeat domain containing proteins may prove exceedingly useful for developing novel rational therapy for cardiac disease and muscle dystrophies.

## Introduction to ankyrin proteins

The protein family of ankyrin repeat containing proteins derives its name from the ankyrin polypeptides that serve as anchor proteins and thus constitute critical structural components in the erythrocyte membrane (Bennett, 1978). Since the discovery of this protein more than 30 years ago, ankyrins have emerged as multifunctional proteins, present in a variety of tissues and cell types, including skeletal and cardiac myocytes (Ayalon et al., 2008; Hashemi et al., 2009), neurons, photoreceptors (Kizhatil et al., 2009a; 2009b), and epithelial cells (Kizhatil et al., 2007a; Bennett and Baines, 2001). Although ankyrins fulfil important functions in many cell types, anchoring cytoskeletal components to the intracellular machinery in muscle tissues is especially important. Ankyrins are particularly prominent in contractile tissues and genetic knock-out experiments convincingly demonstrate the importance of ankyrins in this respect (Mohler et al., 2003; 2004; Borzok et al., 2007). This point is further highlighted by the high expression of ankyrins in the muscle types of non-vertebrates (Chen et al., 2001), showing the strong evolutionary pressure that exists on the presence of ankyrin proteins in this type of tissue.

**Figure 1:**
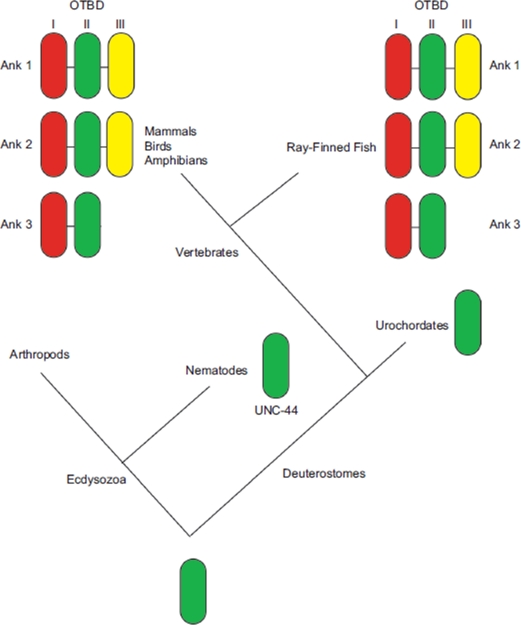
Proposed model of evolutionary events leading to obscurin-titin binding domain (OTBD) in present-day ankyrins. In vertebrates, successive duplications led to three different modules, I, II and III. Ank1 and Ank2 have all three modules, while Ank3 has only modules I and II. Adapted from Hopitzan *et al*. (2006). (permission has been obtained from Oxford University Press for the reproduction on this figure).

In higher vertebrates, there are three canonical ankyrin genes: *Ank1* (Ankyrin-R polypeptides) (Lux et al., 1990), *Ank2* (Ankyrin-B polypeptides) (Otto et al., 1991) and *Ank3* (Ankyrin-G polypeptides) (Kordeli et al., 1995), with only *Ank1* (Lux et al., 1990; Lambert and Bennett, 1993) and *Ank3* (Kordeli et al., 1995; Peters et al., 1995; Kordeli et al., 1998; Thevananther et al., 1998; Mohler et al., 2004) being expressed in the skeletal muscle. The presence of three ankyrin genes is likely due to genome duplications in vertebrates. The nematode *Caenorhabditis elegans* and urochordate *Ciona intestinalis* possess only a single ankyrin gene, while the genome of arthropoda such as *Drosophila melanogaster* contains two ankyrin genes. One view of ankyrin evolution is that they are a solution to the problems of independent motility in metazoans by contributing membrane resilience to the forces of muscle contraction (Bennett and Baines, 2001; Hopitzan et al., 2006). Based on the obscurin-titin binding domain (OTBD), at the C-terminal domain of ankyrins, the Kordeli group described a proposed evolutionary event leading to present day ankyrins (Figure 1) (Hopitzan et al., 2006). Interestingly, a vertebrate-specific module of the OTBD is expressed exclusively in muscle tissues, after the divergence from Urochordates. Following the discovery and resolution of the primary sequence of *Ankyrin* proper, it soon emerged that a variety of other proteins contained one or more repeats of a motif that bear structural resemblance to a stretch of 33 amino acid residues present in the original Ankyrin protein, and was thus named ankyrin repeat (Sedgwick and Smerdon, 1999). The ankyrin repeat is defined by specific shape-determining residues, including a TPLH motif at positions 4 through 7 and glycines at positions 13 and 25, together resulting in the formation of two antiparallel α-helices followed by either a (3-hairpin or a long loop. Such ankyrin repeats were first identified in the sequence of yeast *Swi6p, Chc10p* and Drosophila *Notch* (Breeden and Nasmyth, 1987), and was later named after the cytoskeletal protein Ankyrin as the latter consists of 22 tandem repeats of the 33 amino acid motif (Lux et al. 1990). As ankyrin repeats are present abundantly in a multitude of proteins in all branches of eukaryotic life, the ankyrin repeat as a motif almost certainly predates the ancestral eukaryote living approximately 2.3 billion years ago. The potential of ankyrin repeat proteins to interact strongly with themselves has made this motif exquisitely suitable for functioning as part of a membrane anchor in muscle tissue, explaining the importance of ankyrins for muscle contraction. In addition, it has emerged that the ankyrin motif is present in many other genes expressed in muscle. In the present review, we aim to explore the various functions of the ankyrin repeat domain for skeletal muscle physiology and come to the conclusion that the ankyrin repeat domain is unusually important for the biochemistry of contractile tissue.

## Ankyrin repeat proteins

Following the recognition that a pan-eukaryotic ankyrin repeat motif existed, further investigations have identified a multitude of sometimes very different proteins that display such ankyrin repeats in their primary structure. Often these proteins as a whole, as well as the ankyrin repeats in particular, exhibit strong evolutionary conservation, which is testimony to the versatile action ankyrin repeats can have in cellular function. Indeed, established functions for ankyrin repeat containing proteins are diverse and include regulation of transcription, cell cycle, cell fate determination, cytoskeletal integrity, cellular mechanosensation, and endocytosis (Mosavi et al., 2004). The suitability of ankyrin repeat proteins to act in many diverse physiological settings is dependent on their capacity to interact with other polypeptides, especially with other ankyrin repeats. Furthermore, they are unique in their capacity to be stable both in the highly different redox potential settings of the intracellular and extracellular compartments (Michaely and Bennett, 1992; Sedgwick and Smerdon, 1999). Many investigators have speculated on the importance of this interaction to allow development of complicated multicellular life forms (Marcotte et al., 1999). Ankyrin repeat proteins typically function in mediating specific protein–protein interactions, although recently they have been shown to be required for enzymatic function as well (Rider and Zhu, 2009). A literature search on the cellular roles of ankyrin repeats reveals a strikingly high proportion of muscle-specific publications (12% against e.g. < 4% for PH or SH2 domains), which may be related to the unusual strong nature of ankyrin repeat interactions which can easily survive the mechanical strains of changes in cell shape and the changes in pH and oxidative status that characterizes the muscle cell. An exhaustive screen of the available literature on ankyrin repeat containing proteins in skeletal muscle is given in Table 1, and is subdivided with respect to subclass within the ankyrin repeat superfamily of proteins.

**Table 1:** Ankyrin repeat proteins expressed in skeletal muscle.

Protein	Number of repeats	Organism	Function	Partners	References
*Canonical ankyrins*					
sANK1	2		Linker between sarcomere and sarcomeric reticulum (SR)	Obscurin	11	
ANK3/ANKG	24		SR and post-synaptic membrane organization		19
ANKG119	13		Cell membrane organization and vesicle transport	BIΣ-spectrin	27
*Ankyrin and SOCS box containing proteins*					
ASB2β	11	Mouse	Differentiation	FLNb	28
ASB5	6	Rabbit, mouse	Not known		29; 30
ASB8	4	Human	Not known		31
ASB11	6	Zebrafish	Proliferation and maintenance of muscle progenitor compartment	Ckm?	Unpublished data
ASB15	10, 7	Mouse, human	Protein synthesis, differentiation	Akt	32-34
*Muscle ankyrin repeats*					
Ankrd2	4		Stress response	Titin YB1	35
CARP	4		Stress response	Titin Myopalladin	36
DARP	4		Stress response, energy metabolism	Titin Myopalladin	37
*Other ankyrin repeat proteins*					
Myotrophin	3	Rat	Intitiation of muscle hypertrophy	Actin capping protein; NFKB	38
Notch	7		Muscle differentiation	SKIP	39
βCAP73	6	Bovine	Cell motility		40
NFKB			Inflammation		
Tankyrase2	24	Human	Cytoplasmic signal transduction	Grb14	41

*Ank1*, *sAnk1* and *Ank3* are members of the ankyrin superfamily, which is composed of proteins that are ubiquitously expressed and typically found within the membrane associated cytoskeleton. *Ankg119*, a small cytoplasmic ankyrin isoform, is also important for vesicle transport. Less is known regarding the roles of Asb family of proteins in muscle development, although various ASB proteins are found to be expressed in the skeletal muscle. The most well-studied muscle-related ankyrin repeat proteins are, as clearly suggested by their name, the muscle ankyrin repeat proteins (MARPs), which are generally important for stress response. While the approximately 50% sequence homology between the three different MARP proteins is relatively high, the tissue distribution of the MARPs is different – *Cardiac Ankyrin Repeat Protein (CARP) is highly expressed in cardiac muscle, while Ankyrin Repeat Domain Protein 2, (ANKRD2) and Diabetes Related Ankyrin Repeat Protein (DARP)* are most prominently expressed in skeletal muscle – and there is no upregulation or compensation by the remaining MARPs when one or more are removed (Barash et al., 2007). The question as to the *in vivo* functional redundancy of the three genes, therefore, remains unclear. The possible functions and importance for muscle-expressed members of the superfamily of ankyrin repeat domain containing proteins (which also include the Notch protein) will be the subject of this review, the order of the proteins described following the course of their expression during myogenesis.

## Skeletal muscle development

**Figure 2:**
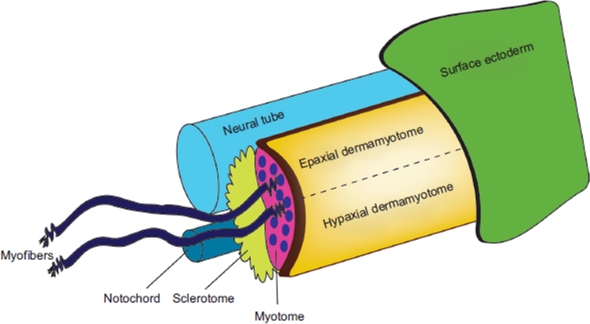
Caricature showing the structures in the skeletal muscle. In general, the main skeletal muscle anatomy consists of the dermomyotome, myotome and sclerotome, and is conserved throughout species. The dermomyotome is the source of the primary myotome, as well as contributing to the formation of the dermis, the endothelial and smooth muscle cells. The dermomyotome is divided into epaxial and hypaxial domains, which give rise to the epaxial muscle (deep muscle of the back) and hypaxial muscles (appendicular musculature, abdominal muscles, diaphragm, hypoglossal chords) respectively.

Skeletal muscle progenitor cells arise from the paraxial mesoderm, which forms the somites. Somites are formed sequentially as segments of the paraxial mesoderm on each side of the neural tube, from anterior to posterior, at regular time intervals. Somites are transient structures that later differentiate into different types of tissue giving rise to several trunk structures: sclerotome (precursor of the bones, cartilages and tendons), myotome (precursor of muscle) and dermatome (precursor of the dermis) (Figure 2; Brand-Saberi and Christ, 2000). The primary myotome is formed as the first differentiated muscle from the dermomyotome between E11.5 and E15.5 in the mouse. At this stage, some myoblasts irreversibly exit the cell cycle, align with each other, and fuse, forming multinucleated myotubes. After primary myogenesis, secondary myoblasts in the dermomyotome use the primary myotome as a scaffold to attach to and fuse with each other, giving rise to secondary myotubes (Bryson-Richardson and Currie, 2008). A similar molecular process of myogenesis occurs postnatally, to recruit adult muscle precursors into forming new myofibers during skeletal muscle damage.

The genetic basis for muscle formation and the signaling pathways involved in patterning the myotome is similar in all vertebrates. Primary myogenesis is initiated by signals from the notochord (Sonic hedgehog), neural tube (Wnt) and overlying surface ectoderm (fibroblast growth factor) that induce (Linker et al., 2003) or downregulate (Hirsinger et al., 2001) the expression of the basic-helix-loop-helix myogenic regulatory factors: *Myogenic factor 5* (*Myf5*), *Myogenic factor 6* (*Myf6*, also known as *Mrf4*), *Myogenic differentiation* (*MyoD*), and *Myogenin* (*Myog*) (Charge and Rudnicki, 2004). Several ankyrin repeat proteins have been shown to negatively regulate these myogenic regulatory factors, and will be described below.

## Notch intracellular domain (Notch ICD)

Notch is emerging as an important molecule in organogenesis. Very broadly, it can be stated that it acts to stimulate proliferation in progenitor compartments whilst simultaneously inhibiting/delaying differentiation. Briefly, upon activation of the Notch ligand, the Notch intracellular domain (Notch ICD) is cleaved and released from the plasma membrane and translocates into the nucleus to function as a transcriptional coactivator for CSL (mammalian C promoter-binding factor 1, also known as CBF1, Suppressor of Hairless or LAG1) proteins (reviewed in Weinmaster, 2000; Fortini, 2001; 2002). Notch inhibits myogenesis caused by Myf5 or MyoD (Kopan et al., 1994) or inhibits DNA binding by Mef2c and the cooperation of Mef2c for Myod and Myogenin DNA binding (Wilson-Rawls et al., 1999). The ankyrin repeat located in the ICD of Notch plays a significant role in these inhibitory actions for myogenesis (Kopan et al., 1994; Wilson-Rawls et al., 1999). Thus, Notch ICD is a crucial determinant of compartment size in pregestational muscle.

It is important to note that proper Notch-induced gene expression in many cases involves a process called lateral inhibition. Once Notch signaling is initiated by its ligands of the Delta family, Delta is downregulated in the Notch signaling cell. In turn, this causes diminished Notch signaling in the neighboring cells, which react to upregulate Delta, amplifying differences between adjacent cells. Powerful negative feedback mechanisms, however, act on this lateral inhibition and a substantial original bias is essential for Notch signaling to ensue. Recent work showed that in the developing nervous system especially, the subfamily of six ankyrin repeat domain containing ASB proteins is important for creating the original bias that allows lateral inhibition to develop (Diks et al., 2008). We discuss below the importance of ASB proteins in later stages of muscle development, as they control important steps of muscle cell differentiation, probably at least partly though control of Notch signaling.

## Ankyrin repeat and SOCS box containing proteins (ASB)

The ankyrin repeat and SOCS box (ASB) family of proteins contains two functional domains: an ankyrin repeat region where specific protein–protein interactions occur, and a SOCS box region, which serves as a generic adaptor directing the degradation of proteins targeted by the ankyrin repeat region (Kile et al., 2002). To date, 18 mammalian ASB proteins have been identified. These 18 ASB proteins have varying forms and numbers of ankyrin repeats and other novel regions, suggesting they bind different target proteins (Li et al., 2007). Human ASB3 and ASB8 proteins are strongly expressed in the skeletal muscle (Liu et al., 2003; Chung et al., 2005), while ASB6, ASB7 and ASB9 proteins are weakly expressed in the skeletal muscle (Human Protein Atlas). In mice, Asb2, Asb5, Asb8, and Asb10 proteins are strongly expressed in the skeletal muscle (Kile et al., 2000; 2001). Interestingly, Asb5 was found to be expressed in both quiescent and activated satellite cells (Boengler et al., 2003; Seale et al., 2004), as well as three days after differentiation (Seale et al., 2004). Although it has been known for some time that ASB proteins are expressed in skeletal muscle, the important functional role of ASBs in skeletal myogenesis has only recently received recognition. Different ASBs, however, have markedly different actions in muscle development, maybe as a consequence of the different number of ankyrin repeats these proteins contain.

*Myotrophin/V-1* is evolutionarily conserved and is expressed at low basal levels in every mammalian organ and cell type (Sivasubramanian et al., 1996b; Anderson et al., 1999) with the least expression in skeletal muscle (Sivasubramanian et al., 1996b). The levels of Myotrophin/V-1 were found to be elevated in tissues of failing human hearts (Sil et al., 1993), although the levels of these proteins gradually decreased in human plasma during the progression of heart failure (O’Brien et al., 2003). Myotrophin/V-1 has been shown to stimulate protein synthesis in cardiomyocytes leading to hypertrophy, as well as the expression of a number of cardiac genes (e.g*. β-myosin heavy chain* and *atrial natriuretic peptide*) and proto-oncogenes (e.g. *c-Myc*, *c-Fos* and *c-Jun*) (Mukherjee et al., 1993; Sivasubramanian et al., 1996a; Hayashi et al., 2001; Gupta et al., 2002; Gupta and Sen, 2002; Knuefermann et al., 2002; Mosavi et al., 2002) and hence seems to play an important role in muscle adaptation to increased load. How this relates to its functions in non-muscle tissue, however, remains unclear.

Myotropin/V-1 resembles a truncated form of I-κBα protein without the signal response domain, nuclear localization signal masking domain and PEST degradation domain (Knuefermann et al., 2006). The ankyrin repeats in Myotrophin/V-1 are capable of interacting with the rel domain of NF-κβ protein, which is also an ankyrin repeat containing protein itself (Knuefermann et al., 2002). Several studies have proposed that Myotrophin/V-1 is an extracellular growth factor, which functions to initiate cell surface signal transduction events leading to cardiac hypertrophy (Sen et al., 1990; Sil et al., 1998). Contrasting studies however show that extracellular expression of Myotrophin/V-1 does not provoke hypertrophy (Pennica et al., 1995; Yamakuni et al., 2002), and that its function is mainly intranuclear, acting as a modifier of NF-κB in the nucleus, possibly by promoting the formation of *Rel* family homodimers over heterodimers. As NF-κB activation is a predicted response to challenging muscle load, it is easy to envision how such a nuclear function could be implicated in the regulation hypertrophic response. Also, the presence of a nuclear localization signal and the absence of a clear secretion signal (as is present in insulin-like growth factor-1, to which in the original publications on the extracellular functioning of Myotrophin/V-1 the protein was compared), we strongly favor the nuclear hypothesis, although definitive experiments that include the introduction of *Myotrophin/V1* variants that lack the nuclear localization domain could help provide the final answer here (Gupta et al., 2002; Knuefermann et al., 2006).

Although the activity of Myotrophin/V-1 as a hypertrophic molecule in cardiac muscle is fairly well established, there is less known regarding the role of Myotrophin/V-1 in skeletal muscle. A study showed that exogenous application of Myotrophin/V-1 to skeletal muscle cells has hypertrophic effects, suggesting that the protein has at least the potential to act as hypertrophic molecules in such tissue. However, whether it also functions as such in practice is still a very open question (Hayashi et al., 1998). Expression of *Myotrophin/V-1* in myoblasts decreases during the process of muscle differentiation, reaching an undetectable level in mature skeletal muscle, suggesting that it does not have a major physiological role in this context. In contrast, the expression of *Myotrophin/V-1* is markedly increased in regenerating muscles of Duchenne muscular dystrophy and of its animal model, *mdx* mouse (Furukawa et al., 2003). Thus, further work is necessary to address this issue.

## Muscle ankyrin repeat proteins (MARPs)

There are three identified proteins in the family of muscle ankyrin repeat proteins (MARPs): CARP/MARP, ANKRD2/ARPP, and DARP. All three molecules were identified previously by their cytokine-like induction following cardiac injury and muscle denervation (CARP/MARP) (Baumeister et al., 1997; Kuo et al., 1999; Aihara et al., 2000), skeletal muscle stretch (ANKRD2/ARPP) (Kemp et al., 2000), or during recovery after metabolic challenge (DARP) (Ikeda et al., 2003). These three isoforms share in their C-terminal region a minimal structure composed of four ankyrin repeats involved in protein–protein interaction, PEST motifs characteristic of proteins targeted for rapid degradation protein, and at the N-terminal region, a putative nuclear localization signal (Miller et al., 2003; Lydie et al., 2009). The members of this nuclear as well as cytoplasmic family of proteins (Zou et al., 1997; Ishiguro et al., 2002; Tsukamoto et al., 2002) are found in the central I-band of the sarcomeres, where they bind to the N2A region of *Titin* (Miller et al., 2003), and the amino terminus of Nebulin anchoring protein, myopalladin (Bang et al., 2001). The Titin-binding domain is located in the second ankyrin repeat of all three proteins (Miller et al., 2003). Their function is as a resource of last resort to maintain muscle function despite high demands; this is supported by their induction following strain and muscle injury, and their capacity to reinforce muscle structure through interaction with structural elements of contractile machinery, by introducing the highly robust pH- and redox-insensitive ankyrin bonds as a response to excessive demand to the muscle tissue. In agreement with this notion, mice lacking all three MARP proteins show a relatively mild phenotype, with a trend towards a slow fiber type distribution, but without differences in muscle fiber size (Barash et al., 2007) Thus, the expression of this family of ankyrin repeat domain containing proteins suggests that this family is a part of the machinery that helps muscle cells deal with excessive mechanical load.

CARP, also known as C-193, was originally isolated as a cytokine responsive gene in fibroblasts (Chu et al., 1995), but its main action seems to lie in the heart, where it helps in controlling hypertrophic reactions by providing negative feedback to the genomic cardiac hypertrophic response. However, as a cytoplasmic structural protein, it reinforces the cardiac contractile machinery, a response which acts to limit the consequences of excessive demand on heart pump function. Support for this view comes from the observation that it is naturally upregulated during hypertrophy and downregulated during atrophy, and that aberrant upregulation of this protein can actually drive atrophy under certain conditions (Baumeister et al., 1997; Stevenson et al., 2003; Yang et al., 2005). CARP is expressed throughout all the heart chambers. Furthermore, the protein is also present, albeit much more weakly expressed, in skeletal muscle (Ishiguro et al., 2002; Tsukamoto et al., 2002) where it probably serves similar functions, although this has been less well investigated. Its possible beneficial role as a cardiac anti-hypertrophic mediator over the past 15 years has prompted a significant research effort into this protein. As to be expected from such an anti-hypertrophic gene, CARP inhibits cardiac-specific gene expression and hence its expression is differentially regulated between embryonic and adult heart (Baumeister et al., 1997; Zou et al., 1997; Jeyaseelan et al., 1997; Kuo et al., 1999), as to allow proper cardiogenesis. The protein has both a nuclear and a cytoplasmic action. Its role in the negative feedback on cardiac hypertrophic genomic responses is dependent on the former form of the protein (Jeyaseelan et al., 1997; Zou et al., 1997), although not all mechanistic details as to how nuclear CARP influences gene expression have been elucidated. Overexpression in cardiomyocytes results in suppression of *Cardiac troponin C* and *Atrial natriuretic factor* transcription (Jeyaseelan et al., 1997). CARP interacts with integral components of the muscle such as desmin and titin (Mikhailov and Torrado, 2008). CARP also interacts with the transcription factor YB1 and inhibits the synthesis of the ventricular specific myosin light chain 2v (MLC-2v) (Zou et al., 1997).

As stated, the molecular function of CARP in skeletal muscle is less well known, but there it also seems mainly to act in limiting the consequences of excessive load. Recently, it has been suggested that CARP is important for sarcomere length stability and muscle stiffness, as well as having an inhibitory role in regenerative responses of muscle tissue (Barash et al., 2007). CARP overexpression induces a switch towards fast-twitch muscle fibers (Lydie et al., 2009). Interestingly, CARP was found to be expressed exclusively in small regenerating myofibers in muscular dystrophy patients (Nakada et al., 2003b) as well as significantly upregulated in numerous muscular dystrophy models and denervation induced atrophy (Lydie et al., 2009). In vascular smooth muscle cells, increased CARP expression has been demonstrated to be associated with upregulation of the protein p21WAF1/CIP1, an inhibitor of the cell cycle (Kanai et al., 2001), which might also be seen as a protective response. Thus, CARP as protein involved in limiting damage to muscle overactivation does not show an absolute restriction towards the skeletal muscle lineage.

Like CARP, *Ankrd2* acts to limit damage following excessive demand on muscle, and accordingly, it was first identified as a stretch responsive gene product upregulated in stretched muscle (Kemp et al., 2000). *Ankrd2* expression is not easily induced, with upregulation only seen under eccentric contractions, while most other muscle proteins such as MyoD, Myogenin, Muscle LIM protein and CARP are sensitive to mechanical strain under both isometric and eccentric contractions (Barash et al., 2004; Hentzen et al., 2006). Thus, *Ankrd2* induction seems to be a protection mechanism of last resort. *Ankrd2* shows a distinctive preference for expression in slow skeletal fibers and cardiac atria (Pallavicini et al., 2001; Kojic et al., 2004). Ankrd2 interacts with transcription factors YB-1, PML and p53 (Pallavicini et al., 2001; Kojic et al., 2004), and is localized to PML bodies in proliferating myoblasts where it modulates their transcriptional activity. Ankrd2 accumulates in the nuclei of myofibers located adjacent to severely damaged myofibers after muscle injury. It translocates from the I-band to the nucleus after muscle injury, and may participate in regulation of gene expression (Tsukamoto et al., 2008). Hence, unlike CARP, it only acts on the transcriptional level and thus does not serve as a structural component, maybe because other ankyrin repeat containing proteins are induced at lower levels of muscle stress and occupy the available binding sites for such proteins in the contractile machinery.

The least studied MARP is *DARP*, which is expressed in both heart (low expression) and skeletal muscle (high expression). It was identified by its upregulation in Type 2 diabetes and insulin-resistant animals. Thus, DARP has been implicated with a potential role in energy metabolism (Ikeda et al., 2003). Similar to CARP, DARP interacts with Titin-N2A and Myopalladin (Miller et al., 2003).

## Application of ankyrin repeat proteins in muscle disease

As evident from the above, the different stages of muscle development and their phenotypic reaction to strain and exercise are under the control of different ankyrin repeat domain containing proteins, and accordingly their expression at different stages of muscle development seems to be tightly regulated. This offers the obvious possibility that manipulating such expressions may be useful for dealing with muscle diseases. These hopes are especially fostered now that adeno-associated virus gene therapy introduced in human muscle has proven both safe and useful for the treatment of lipoprotein lipase (LPL) deficiency (Mingozzi et al., 2009) and thus introduction of specific ankyrin repeat containing proteins into patients is certainly technically and ethically feasible.

Muscle diseases such as muscular dystrophies or inherited myopathies have a general characteristic of progressive muscle weakness and degeneration. In the past decade, great advances have been made in clinical studies of muscle disease. The most recent advances in clinical and experimental studies of muscle diseases, such as muscular dystrophies and related myopathies, as well as the state of our present knowledge on these diseases have been recently reviewed in Cardamone et al. (2008) and Willmann et al. (2009). In a recent gene expression profiling of patients in the pre-symptomatic phase of Duchenne muscular dystrophy (DMD), altered expression of more than 30 ankyrin repeat proteins was identified (Pescatori et al., 2007), which makes sense in view of the importance this family of proteins has in dealing with stress and damage to muscle tissue. Of special interest is the recent discovery that ankyrin proteins (ankyrinB and ankyrinG) bind to dystrophin and dystroglycan respectively, and are required for the retention of these proteins at the costameres (Ayalon et al., 2008), further highlighting the importance of the ankyrin repeat domain in the context of the demands muscle physiology makes on protein–protein interactions. This point is especially vividly illustrated by a Becker muscular dystrophy mutation, which reduces ankyrinB binding and impairs sarcolemmal localization of dystrophin-Dp71 (Aartsma-Rus et al., 2006; Ayalon et al., 2008), causing disease; thus demonstrating the deleterious muscle-specific consequences of ankyrin bonding failing to occur. In line with the role of ankyrin repeat domain proteins as a last line of defense against excessive muscle load is the increase in CARP expression with an array of muscle pathologies: DMD, spinal muscular atrophy, facio-scapulo-humeral muscular dystrophy, amyotrophic lateral sclerosis, and peroxisome proliferator-activated receptor induced myopathy (Nakada et al., 2003a; Casey et al., 2008), as well as the *mdx*, Swiss Jim Lambert (SJL) and muscular dystrophy with myotitis (MDM) animal models, deficient respectively in dystrophin, dysferlin and titin (Bakay et al., 2002; Nakamura et al., 2002; Porter et al., 2002; Nakada et al., 2003a; 2003b; Witt et al., 2004; Suzuki et al., 2005). Expression of MARP is reduced in dystrophic muscle (Pallavicini et al., 2001) but increased following denervation (Tsukamoto et al., 2002), in a mouse model of muscular dystrophy with myositis due to titin N2A deletion and in heart failure (Zolk et al., 2002). Thus, human muscle diseases highlight the special importance of the ankyrin bond for muscle physiology.

In apparent agreement with such a role, the functional deficiency of ankyrin repeat containing protein in skeletal muscle is not limited to muscle degenerative diseases. Ankrd2 was detected in approximately 90% of rhabdomyosarcoma tissues but only when accompanied by morphological evidence of skeletal muscle differentiation of tumor cells (Ishiguro et al., 2005), suggesting that in the context of neoplastic dedifferentiation, expression of ankyrin repeat proteins correlates with functionality. It would be interesting to investigate the extent to which expression of such protein is sufficient to counteract dedifferentiation. In any case, the fact that all these muscle abnormalities are associated with expression of specific ankyrin repeat domain proteins fits well with the notion that developmental programs are controlled (and structural elements also partially affected) by expression of specific ankyrin repeat domain proteins. On a related note, the high binding affinity of ankyrins in strengthening and maintaining the skeletal muscle structure suggests that the induction of ankyrin repeat containing proteins in skeletal myopathies may play a role in the survival of the diseased muscle fibers. Many clinical conditions such as heart failure, inflammatory myopathies, chronic arthritis, and aging are associated with muscle wasting and weakness. Furthermore, elderly or bed-ridden patients and space travelers undergoing long periods of muscle disuse often show signs of muscle weakness and atrophy. While myostatin (Sharma et al., 2001; Zimmers et al., 2002) and glucocorticoids (Tischler, 1994) have been studied for a role in atrophy, and both can induce atrophy in normal muscle, neither is required for disuse atrophy *in vivo* (Tischler, 1994; McMahon et al., 2003). Kadarian and Hunter recently showed *in vivo* that inhibition of the ankyrin repeat containing proteins Bcl-3 and NF-κB1 prevents muscle atrophy (Hunter and Kandarian, 2004). Now that the clinical tools that allow temporary expression of proteins in humans are becoming available, it can be envisioned that patients after long bed rest and muscle disuse might be treated by expression of muscle strengthening ankyrin repeat domain proteins in the most important skeletal muscles to aid rehabilitation.

## Concluding remarks

**Figure 3:**
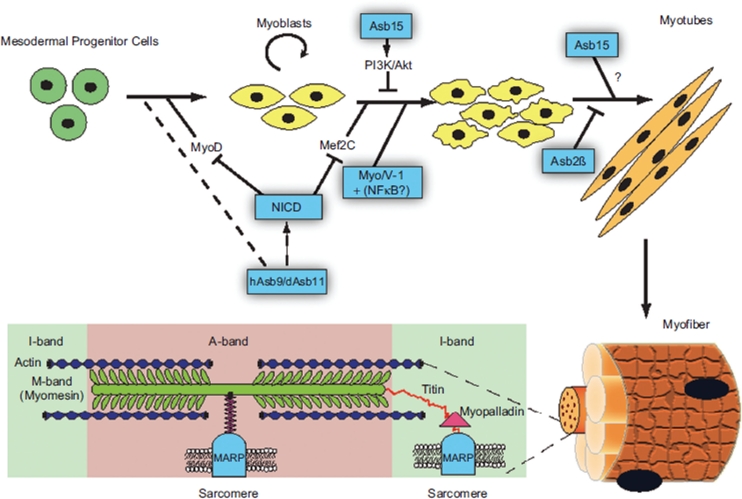
Summary of the ankyrin repeat proteins in muscle biology, from specification and differentiation of muscle precursors (hAsb9/dAsb11, NICD, Asb15, Myo/V-1+NFKB?, Asb2β) to the structures of the muscle fibers (MARPs).

Although the ankyrin bond has a general importance for vertebrate cellular biochemistry and physiology, its specific properties have led to it acquiring specific functions in muscle biology (Figure 3). The specific expression of different ankyrin repeat domain containing proteins during the various phases of muscle development allows this module to mediate specialized functions during muscle development. Obviously this allows for a high level of regulation, but also offers the opportunity for clinical use during muscle specific disease. We predict that further research will further reveal unique functions for ankyrin repeat domain containing superfamily members in muscle cell physiology.

## Declaration of interest

JMT was funded by ALW Grant #81502006. MPP receives funding from TI-Pharma and ALW Grant #81702002. The authors report no conflicts of interest.
